# Hierarchically Structured CeO_2_ Catalyst Particles From Nanocellulose/Alginate Templates for Upgrading of Fast Pyrolysis Vapors

**DOI:** 10.3389/fchem.2019.00730

**Published:** 2019-10-30

**Authors:** Kathleen Moyer, Davis R. Conklin, Calvin Mukarakate, Derek R. Vardon, Mark R. Nimlos, Peter N. Ciesielski

**Affiliations:** ^1^Interdisciplinary Materials Science Program, Vanderbilt University, Nashville, TN, United States; ^2^National Renewable Energy Laboratory, National Bioenergy Center, Golden, CO, United States; ^3^National Renewable Energy Laboratory, Biosciences Center, Golden, CO, United States

**Keywords:** nanocellulose (NC), catalysis, fast pyrolysis, hierarchical structure, templated synthesis

## Abstract

Hierarchically structured porous materials often exhibit advantageous functionality for many applications including catalysts, adsorbents, and filtration systems. In this study, we report a facile approach to achieve hierarchically structured, porous cerium oxide (CeO_2_) catalyst particles using a templating method based on nanocellulose, a class of renewable, plant-derived nanomaterials. We demonstrate the catalyst performance benefits provided by this templating method in the context of Catalytic Fast Pyrolysis (CFP) which is a promising conversion technology to produce renewable fuel and chemical products from biomass and other types of organic waste. We show that variations in the porous structures imparted by this templating method may be achieved by modifying the content of cellulose nanofibrils, cellulose nanocrystals, and alginate in the templating suspensions. Nitrogen physisorption reveals that nearly 10-fold increases in surface area can be achieved using this method with respect to commercially available cerium oxide powder. Multiscale electron microscopy further verifies that bio-derived templating can alter the morphology of the catalyst nanostructure and tune the distribution of meso- and macro-porosity within the catalyst particles while maintaining CeO_2_ crystal structure. CFP experiments demonstrate that the templated catalysts display substantially higher activity on a gravimetric basis than their non-templated counterpart, and that variations in the catalyst architecture can impact the distribution of upgraded pyrolysis products. Finally, we demonstrate that the templating method described here may be extended to other materials derived from metal chlorides to achieve 3-dimensional networks of hierarchical porosity.

## Introduction

**Figure 1:**
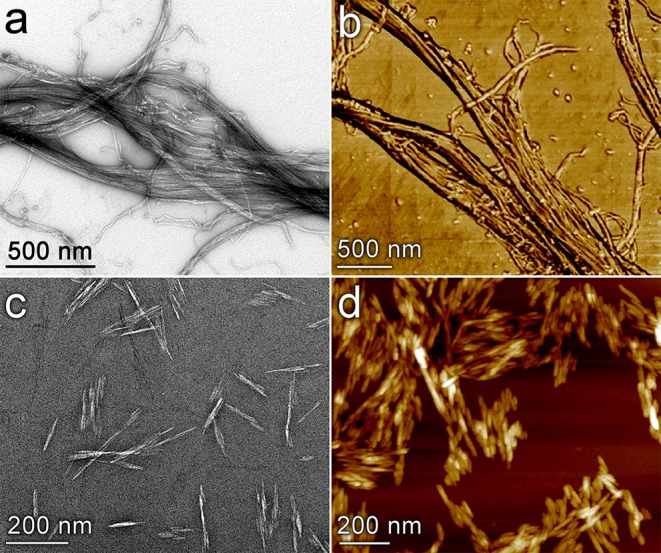
Images of nanocellulose materials used as mesoporous catalyst templates. Cellulose nanofibrils **(a,b)** and cellulose nanocrystals **(c,d)** are visualized by transmission electron microscopy **(a,c)** and atomic force microscopy **(b,d)**. The image in **(b)** is a phase image; **(d)** is a topology image.

The development of rapid, scalable, and environmentally sustainable methods to produce hierarchically structured porous materials can provide substantial benefit to many industrial processes due to the importance of these materials in applications such as catalysis, separations, and energy storage (Yang X.-Y. et al., 2017). Recent studies have highlighted the importance of controlling porosity of materials over multiple length scales in order to optimize transport phenomena coupled to adsorption/desorption processes and chemical reactions in filtration (Xu et al., 2014; Wisser et al., 2015; Liu et al., 2019) and catalytic applications (Feliczak-Guzik, 2018; Sierra-Salazar et al., 2018; Bharadwaj et al., 2019). Commonly employed strategies to produce these materials use templating methods that rely upon the self-assembly of surfactants, polymers, and nanomaterials (Yang X.-Y. et al., 2017). Nanocellulose (NC) materials are ideal candidates for such templating purposes for a variety of reasons, including their low cost (Nelson et al., 2016), tunable size and morphology (Brinchi et al., 2013), readily-modified surface chemistry (Habibi, 2014), large production capacity (Shatkin et al., 2014), controllable self-assembly behavior (Habibi et al., 2010), potential to be produced in tandem with renewable biofuels (Yarbrough et al., 2017), and environmental compatibility (Endes et al., 2016). The most common and readily available forms of nanocellulose typically fall into two distinct classes based on the size and shape of the particles: cellulose nanocrystals (CNC) which are typically 50–500 nm long with a diameter of 3–20 nm; and cellulose nanofibrils (CNF) which are classified as having a diameter of ~100 nm or less and length of 500 nm or longer and can form an entangled fibril network (Zhu et al., 2016). Figure 1 presents images of cellulose nanofibrils (top row) and cellulose nanocrystals (bottom row) obtained by transmission electron microscopy (left column) and atomic force microscopy (right column). Various forms of NC have been investigated previously for catalyst synthesis applications. Many of these applications have used NC directly as a support for metallic nanoparticles as summarized in a recent review by Kaushik and Moores (2016). Alginate, an abundant polysaccharide derived from brown algae, has also been used to prepare high-surface area catalyst supports (Valentin et al., 2005). Additional studies have used NC as a template to achieve mesoporous materials (Shin and Exarhos, 2007; Cai et al., 2015; Kong et al., 2015; Chen et al., 2016; Yang L. et al., 2017; Shen et al., 2018) and hollow tubular structures (Korhonen et al., 2011; Torres-Rendon et al., 2016; Zhu et al., 2017). Several groups have utilized the ordered self-assembly behavior of negatively charged CNCs to achieve chiral mesoporous materials (Dujardin et al., 2003; Shopsowitz et al., 2010, 2011).

Here, we present a facile fabrication method to prepare hierarchically structured mesoporous materials using combinations of NC and alginate We demonstrate the effectiveness of our approach by preparing mesoporous cerium oxide (CeO_2_) catalysts used for catalytic fast pyrolysis (CFP) which is promising thermochemical conversion technology for producing renewable fuels and chemicals from biomass (Ciesielski et al., 2018). The chemical composition and structural architecture of catalyst used in the pyrolysis process plays a critical role in determining the product slate that results from upgrading of pyrolysis vapors. Researchers have demonstrated that reducible metal oxides are effective at converting oxygenated pyrolysis products into useful monofunctional molecules (Lu et al., 2010). Based on the choice of catalyst, this process can be tuned to produce functional mono-oxygenated molecules, such as ketones and phenolics. Ketones and alcohols are desired monofunctional intermediates as they can undergo further C-O bond cleavage and C-C bond formation from the initial pyrolytic breakdown to yield various types of chemicals and fuels. A recent study by Mante et al. (2015) revealed that pure CeO_2_ exhibits ketonization activity when used to upgrade cellulose pyrolysis vapors. The high catalytic activity of CeO_2_ can be ascribed to its active redox properties involving exchange of Ce^4+^ and Ce^3+^ ions, Lewis basicity, and increased oxygen exchange and storage capacity (Mante et al., 2015). These features of CeO_2_ imply its utility for the production valuable chemicals alongside biofuels in a CFP format which is a central strategy to improve economic viability (Cai et al., 2018). Native CeO_2_ is known to have a high surface area that can effectively transport reactant molecules to active sites (Li et al., 2012); however, the bulk surface area of commercially available CeO_2_ powders is low relative to many engineered catalysts used in industrial applications. The development of simple, tunable synthesis methods for high-surface-area CeO_2_ catalysts remains a challenge (White et al., 2014).

A promising approach for increasing the surface area of CeO_2_ catalysts was offered by Laha and Ryoo (2003) who synthesized structured CeO_2_ nanoparticles using silica templates which resulted in particles with uniform mesoporosity within a nanocrystalline framework. A similar approach was subsequently demonstrated for photocatalytic applications by Ji et al. (2008). While these studies demonstrated that increased surface area could be achieved, the synthesis methods required a premade mesoporous silica template, and the resultant materials lacked higher-order architecture that could serve to further enhance transport of reactants and products within larger catalyst particles. Our objective in the present study is to develop a simple, cost-effective template-assisted synthesis method for hierarchically structured CeO_2_ catalyst particles that provide mesoporosity to enhance surface area in tandem with larger pores and channels to improve long-range transport throughout the catalyst.

## Materials and Methods

### Catalyst Synthesis

**Figure 2:**
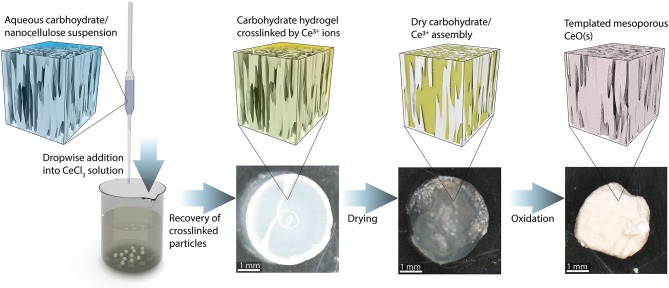
Schematic depiction of the catalyst synthesis process. An aqueous suspension of NC and/or alginate is added dropwise to a solution of CeCl_3_. The negatively charged carbohydrate components are crosslinked by the Ce^3+^ and form roughly spherical particles. The particles are recovered and freeze-dried. The dried particles are heated to 773 K in air to burn off the carbohydrate scaffold and oxidize the Ce^3+^ to CeO_2_ (s).

A schematic illustrating the catalyst the synthesis procedure is presented in Figure 2. The first step consists of preparing an aqueous carbohydrate suspension to serve as the templating material. Several mixtures of CNFs, CNCs, and sodium alginate were prepared. Alginate is frequently used for synthesizing biopolymeric materials and is commonly used in the formation of hydrogel micro particles (Paques et al., 2014). This polysaccharide is negatively charged at neutral pH and is easily crosslinked to form a hydrogel in the presence of di- and trivalent cations; thus, dropwise addition of the carbohydrate mixture containing sodium alginate to a saturated CeCl_3_ solution results in the formation of hydrogel beads crosslinked by Ce^3+^ ions. The bead formation process is similar to that commonly employed to form hydrogels by crosslinking alginate (Valentin et al., 2005), however the trivalent Ce^3+^ used in this study provides the crosslinking function similar to that typically contributed by Ca^2+^ ions. These beads are recovered from the solution and freeze dried to preserve the solution structure of the carbohydrate/Ce^3+^ matrix (De France et al., 2017). The resulting cryogel particles are then placed in a lab oven in air at 773 K for 4 h to burn off the carbohydrate and oxidize the Ce^3+^ to CeO_2_.

Four different templating solutions were prepared to investigate the structural features imparted by each component: (a) 1.0 wt.% alginate, (b) 1.0 wt.% CNC 1.0 wt.% alginate, (c) 1.0 wt.% CNF 1.0 wt.% alginate, (d) 0.5 wt.% CNC 0.5 wt.% CNF 1.0 wt.% alginate. Alginate was included with the NC templating agents to ensure sufficient crosslinking would occur when the carbohydrate suspension was introduced into the CeCl_3_ solution. A total carbohydrate content of ≤ 2 wt.% was used in each case in order to achieve a viscosity of the templating solution that facilitated consistent drop formation from the tip of the pipette. Attempts to use carbohydrate contents higher than 2% in the templating solution resulted in inconsistent flow from pipette tip which resulted in catalyst particles of heterogeneous size and shape. Sodium alginate and cerium (III) chloride heptahydrate (CeCl_3_·7H_2_O, 99.9%), were purchased from Sigma-Aldrich. CeO_2_ powder (Cerium (IV) oxide, powder, 99.995%) for use as a control was also purchased from Sigma-Aldrich. CNF (3 wt.% aqueous suspension, nominal width 50 nm, several 100 microns in length) and CNC produced by sulfuric acid hydrolysis (11.9% aqueous suspension, 5–20 nm width, 150–200 nm length) were purchased from the University of Maine Process Development Center.

### Thermal Gravimetric Analysis (TGA)

To verify complete removal of the carbohydrate template during calcination, TGA was performed on cryogel particles templated with both CNC and CNF. Analysis was performed in static air with a ramp rate of 25°C min^−1^ up to 500°C followed by an isothermal hold at 500°C for 1 h.

### X-Ray Diffraction (XRD)

The crystal structure of the catalyst materials was analyzed by XRD using a Rigaku Ultima IV X-ray diffractometer. The X-ray voltage was set to 40 kV and 44 mA, with a sampling width of 0.02° and scan speed of 5° min^−1^. Metal crystallite sizes were estimated using the Williamson-Hall method with PDXL version 1.6.0.1 (Rigaku Corporation). ICDD PDF card number 01-075-9470 was used as a reference pattern for the cubic fluorite crystal structure of cerium (IV) oxide.

### Scanning Electron Microscopy (SEM) and Energy Dispersive X-Ray Spectroscopy (EDS)

SEM was performed using a FEI Quanta 400 FEG instrument. Samples were mounted on aluminum stubs with conductive carbon tape adhesive prior to imaging. The mounted samples were then sputter-coated with 5 nm of Ir. Images were obtained at accelerating voltages of 10 keV for low magnification and 30 keV for high magnification. EDS was performed using EDAX X-ray detector positioned within the same instrument. Elemental composition was obtained from the EDS spectra using the EDAX Genesis software package.

### Transmission Electron Microscopy (TEM)

To prepare samples for imaging, catalyst particles were suspended in ethanol and drop-cast onto carbon-coated, 200 mesh copper grids (SPI Supplies, West Chester, PA). Grids were allowed to air dry prior to imaging. Imaging was performed using an accelerating voltage of 200 keV, and images were captured with a four mega-pixel Gatan UltraScan 1000 camera (Gatan, Pleasanton, CA) on a FEI Tecnai G2 20 Twin 200 kV LaB6 TEM (FEI, Hilsboro, OR).

### Nitrogen Physisorption

Surface area, pore volume, and average pore diameter were determined by multipoint nitrogen physisorption using a Quantachrome QuadraSorb SI analyzer. Prior to measurement, catalysts were outgassed at 250°C for 18 h. The measurement was performed in liquid nitrogen at −196°C. BET analysis was used to obtain specific surface area, and BJH and t-plot methods were used evaluate pore volume and pore size. Points for BET analysis were selected to include the point of statistical monolayer formation and ensure that the product of the quantity adsorbed with (1–p/p0) was strictly increasing over the range of points included in the calculation (Sing, 2014). All BET fits had *R*^2^ > 0.998. BJH analysis was performed on the adsorption branch of the isotherm using the Broekhoff-de Boer thickness model and was limited to pore widths >1.7 nm. *t*-plots were created by using the Harkins-Jura thickness model and fitting points with statistical thickness between 0.3 and 0.5 nm (*R*^2^ > 0.998).

### Catalytic Fast Pyrolysis and Tandem Micropyrolyzer GC-MS (py-GC-MS/FID)

To evaluate catalyst performance, all synthesized catalysts and CeO_2_ powder, which served as a control, were tested in a py-GC-MS micropyrolyzer reactor. Experiments were run in triplicate. The py-GC-MS was coupled to a tandem micropyrolyzer (Rx-3050TR, Frontier Laboratories, Japan) equipped with an auto sampler (AS-1020E) and a microjet cryo-trap (MJT-1030Ex). A detailed description of this system was provided in previous studies (Mukarakate et al., 2014; Xu et al., 2017; Stanton et al., 2018). Briefly, 500 μg of cellulose was loaded in deactivated stainless-steel cups followed by a layer of 10 mg catalysts. The cellulose and catalyst layers were separated by quartz wool. The whole sample cup assembly was loaded into a furnace maintained at 500°C to pyrolyze biomass and pass the generated pyrolysis vapors through the catalyst fixed bed for upgrading. Subsequently, the upgraded vapors were captured using a liquid nitrogen trap (−196°C, housed inside the GC oven) and desorbed into the inlet of the gas chromatograph (7890B, Agilent Technologies, USA) interfaced with the MS (5977A, Agilent Technologies, USA). The GC oven was programmed to hold at 40°C for 3 min followed by a ramp to 240°C at a rate of 6.0°C min^−1^. The trapped gases were separated by a capillary column (Ultra Alloy-5, Frontier Laboratories, Japan) with a 5% diphenyl and 95% dimethylpolysiloxane stationary phase. Gases (CO and CH_4_) not captured by the −196°C liquid N_2_ trap were sent to the thermal conductivity detector (TCD) via a separate column (GS-GASPRO) for quantification. The oven was programmed to hold at 40°C for 3 min followed by heating to 300°C. The separated pyrolysis vapors were identified using the NIST GCMS library and quantified using an FID (Budhi et al., 2015; Xu et al., 2017; Stanton et al., 2018). Standard calibrations were checked on a weekly basis by comparing FID areas of at least two compounds. The calibration standards consisted of 30 representative compounds (8 aromatic hydrocarbons, 10 oxygenates, 5 olefins, 5 paraffins, CO, and CO_2_). For compounds without standards, the calibration for a compound with similar functional groups of similar molecular weights (C number) was used.

## Results

### Catalyst Characterization

**Figure 3:**
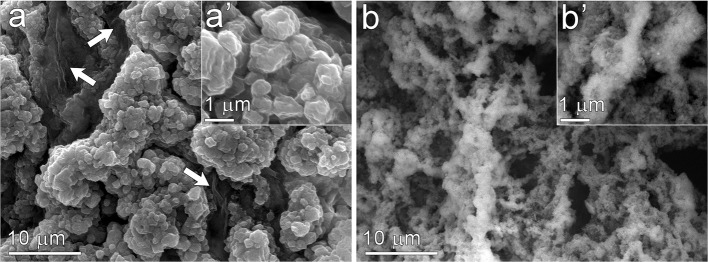
Scanning electron micrographs of the CeO_2_ catalyst templated by CNF, CNC, and alginate before **(a,a')** and after **(b,b')** removal of the carbohydrate template by high temperature oxidation. Arrows in **(a)** indicate carbohydrate domains likely contributed by bundles of CNF.

Scanning electron microscopy (SEM) was used to visualize the structure of the material before and after removal of the carbohydrate template by high temperature oxidation (Figure 3). Images of the CeCl_3_/carbohydrate cryogel following the freeze-drying step but prior to high-temperature oxidation are presented in Figures 3a,a'. These images reveal clusters of predominantly inorganic material that are separated by large domains of carbohydrate (indicated by arrows in Figure 3a) that likely originated from the bundles of cellulose fibrils present in templating solution. Images of the CeO_2_ following removal of the carbohydrate template (Figures 3b,b') reveal a markedly different structure. The large carbohydrate domains observed prior to oxidation are absent, leaving behind channels and void regions within the material. The CeO_2_ clusters shown in Figure 3b' also exhibit a different nanostructure from the aggregates observed in Figure 3a', and appear lacey and porous. These features likely arise from the removal of the small cellulose nanocrystals and single-chain polysaccharides that were promoting gelation of the templating solution in the presence of Ce^3+^ ions.

**Figure 4:**
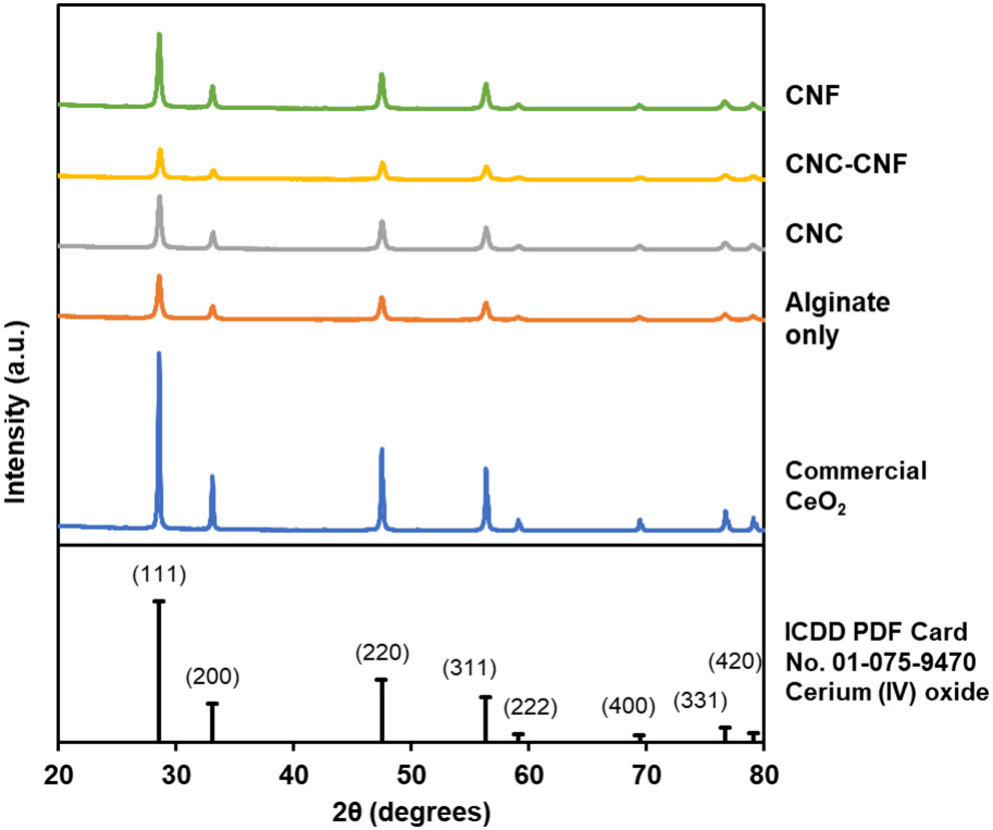
XRD patterns of templated CeO_2_ catalyst particles and commercial CeO_2_ powder confirm that the templated material consists of polycrystalline CeO_2_ in the cubic fluorite crystal structure.

Complete removal of the carbohydrate template from cyrogels during high-temperature oxidation was confirmed by TGA, with a major mass loss around 180°C and insignificant change during a 1 h hold at 500°C. These data are included in the Figure S1. The crystal structure of the templated catalysts was investigated by XRD to confirm that the Ce^3+^ within the carbohydrate cryogel was converted to CeO_2_ by the oxidation processes. The XRD pattern of each type of templated catalyst, along with that of commercial CeO_2_ powder and an ICDD reference pattern, are presented in Figure 4. The measured patterns all clearly exhibit diffraction peaks produced by CeO_2_ in the cubic fluorite crystal structure, with relative peak intensities all closely matching the ICDD pattern (Chelliah et al., 2012). The peak widths of the templated catalysts are wider than those of the commercial powder which is indicative of increased polycrystallinity (i.e., smaller crystal grain size) in the templated materials relative to the non-templated control. Mean crystallite sizes were determined using the Williamson-Hall method to quantify this observation and are presented in Table 1. Templated samples exhibit crystallites roughly 50% smaller than the commercial powder.

**Table 1:** Material properties of commercial and templated CeO_2_.

**Templating agent**	**Surface area (m^**2**^ g^**−1**^)**	**Mesopore volume (cm^**3**^ g^**−1**^)**	**Mean crystallite size (nm)**
None-commercial	3	0.0056	48
Alginate	26	0.0677	21
CNC	27	0.0889	24
CNF	23	0.0819	25
CNC-CNF	28	0.0764	21

*Surface area and mesopore volume are from BET and BJH analysis of nitrogen physisorption data, respectively. Mean crystallite size is from Williamson-Hall analysis of XRD patterns*.

**Figure 5:**
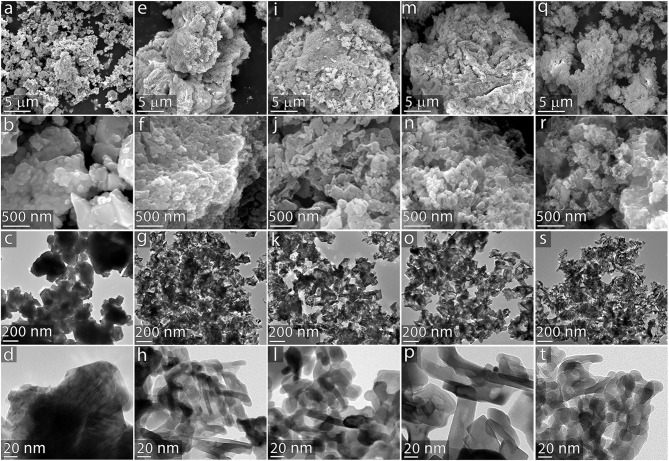
Multiscale electron microscopy of catalyst particles. CeO_2_ catalysts from the following templating agents: **(a–d)** commercially available powder control; **(e–h)** 1.0 wt.% alginate; **(i–l)** 1.0 wt.% CNC 1.0 wt.% alginate; **(m–p)** 1.0 wt.% CNF 1.0 wt.% alginate; **(q–t)** 0.5 wt.% CNC 0.5 wt.% CNF 1.0 wt.% alginate. Images on the top two rows were obtained via SEM; images in the bottom two rows were obtained via TEM.

The multiscale structural features resulting from the variations in templating agents were examined using SEM and transmission electron microscopy (TEM). These results are summarized in Figure 5. SEM images reveal that the commercially available CeO_2_ powder (Figures 5a–d) is relatively smooth in comparison to the templated samples. Images of the material templated by a 1.0 wt.% alginate solution (Figures 5e–h) show that this template introduces mesoporosity into the CeO_2_ material; however, the material does not contain a substantial population of larger channels like those displayed by the particles that were templated with NC components. The introduction of nanocellulose to the templating suspension imparts a controlled, well-defined porosity and surface structure to the CeO_2_ material. The CNC templating agent (shown in Figures 5i–l) introduces fingerlike features to the CeO_2_ catalyst that are larger than those imparted by the alginate alone, resulting in a nanostructured network throughout the material. The nanoscale features here are markedly different than the highly ordered mesoporous assemblies formed by pure suspensions of CNC (Dujardin et al., 2003; Shopsowitz et al., 2010, 2011), which is likely due to a disruption of the self-assembly process by interactions between the CNC and the alginage polymers also present in the templating solution. Particles templated by CNFs (shown in Figures 5m–p) displayed worm-like channels throughout the material. Particles produced by the templating solution containing both CNCs and CNFs (shown in Figures 5q–t) exhibit a combination of features observed in the materials templated by these components individually, consisting of a nanoporous network with larger worm-like channels throughout the structure. These images suggest that the origin of the smaller crystal grain size of the templated catalysts evidenced by the XRD patterns originates from the fingerlike nanostructures observed in Figures 5h,i,p,t.

**Figure 6:**
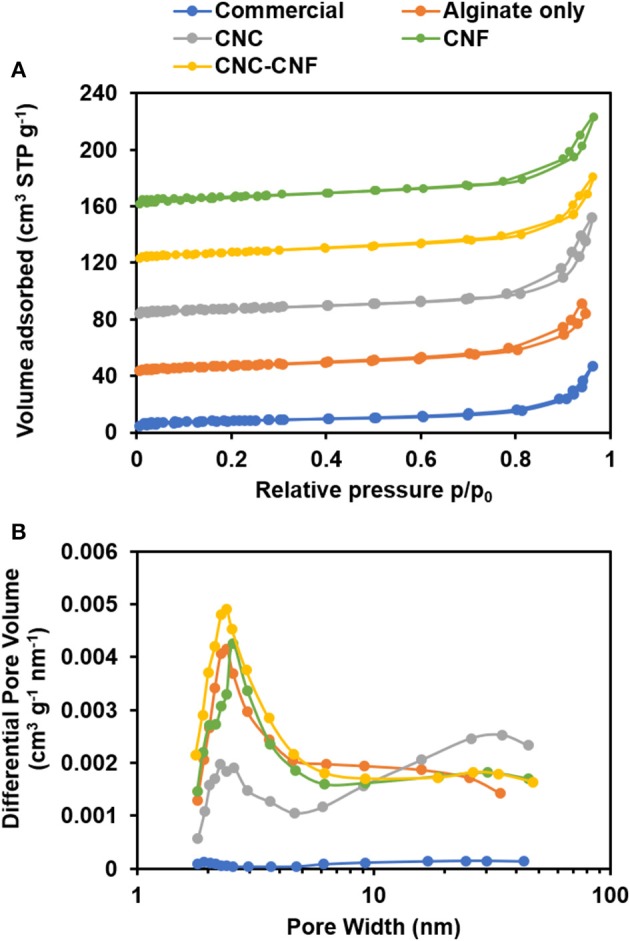
Nitrogen physisorption analysis of CeO_2_ catalyst samples. **(A)** Sorption isotherms reveal the introduction of mesopores into the catalyst structure by the carbohydrate templating. Isotherm of the commercial CeO_2_ powder is scaled up by a factor of 10 and curves are vertically offset by 40 cm^3^ STP g^−1^ to facilitate comparison with other samples. **(B)** The BJH pore volume distribution shows that templates including different carbohydrate components can modify abundance and size of mesopores.

The specific surface area, pore volume, and pore size of the templated materials were characterized by nitrogen physisorption using BET, t-plot, and BJH analysis. Isotherms are presented in Figure 6A. The IUPAC Type II sorption isotherm of the commercial CeO_2_ indicates a lack of mesopores or micropores. In contrast, isotherms of the templated samples exhibit H3-type hysteresis caused by capillary condensation of nitrogen inside mesopores (Sing, 1982). These observations are consistent with SEM and TEM images. All samples show a continuous increase of adsorbed volume as relative pressure approaches one, which is likely caused by macropores and interparticle void spaces observed in SEM images.

BET results revealed that commercial CeO_2_ powder has a surface area of 3 m^2^/g, while the CeO_2_ materials templated with alginate, CNC, CNF, and CNC/CNF templates exhibited a nearly 10-fold enhancement with surface areas ranging from 27–28 m^2^/g. BJH analysis indicates a similar trend in catalyst pore volume, with a greater than 10-fold increase observed for templated samples relative to the commercial sample. The pore volume distribution of each template type is plotted in Figure 6B. Though all of the templated catalysts exhibit a much higher mesopore volume than the commercial sample, the choice of templating solution affects the size of pores which are formed during template burn off. Alginate alone produces a significant amount of smaller mesopores around 2–3 nm in width but is less effective at creating larger pores. The addition of CNC significantly increases the population of larger mesopores around 40 nm width, which may arise due to formation of the finger-like structures seen in TEM images. CNF also causes formation of larger mesopores relative to alginate alone, but the difference is less significant because the major impact of this template is the formation of macropore channels which are too large to be directly captured in this measurement. t-plot analysis (included in Supplemental Material showed that neither the commercial nor templated materials possess micropores.

### Catalytic Upgrading of Cellulose Pyrolysis Vapors

**Figure 7:**
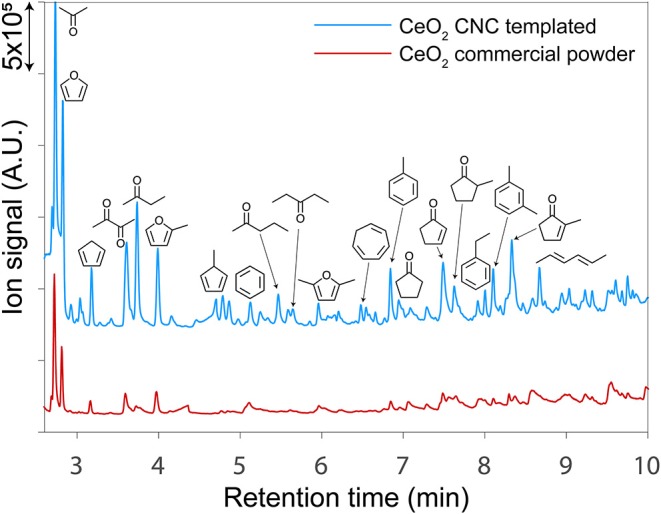
Gas chromatographs from py-GC-MS/FID experiments using a templated CeO_2_ catalyst (blue) and a non-templated CeO_2_ powder (red). The templated catalysts consistently produced substantially higher ion signals which is indicative of their enhanced activity on a gravimetric basis relative to the non-templated catalysts.

The impact of our templating method on catalyst performance was evaluated in the context of upgrading cellulose pyrolysis vapors using CeO_2_ in a py-GC-MS/FID micropyrolyzer reactor. Representative gas chromatographs from py-GC-MS experiments using commercial CeO_2_ powder (red trace) and catalyst templated by a CNC/alginate suspension (blue trace) is presented in Figure 7. As expected, all of the CeO_2_ catalysts produced a suite of predominantly monofunctional small oxygenates; however, the templated catalysts consistently produced substantially higher ion counts during the upgrading experiments which is indicative of their higher overall activity than the non-templated catalyst as visualized in Figure 7.

**Figure 8:**
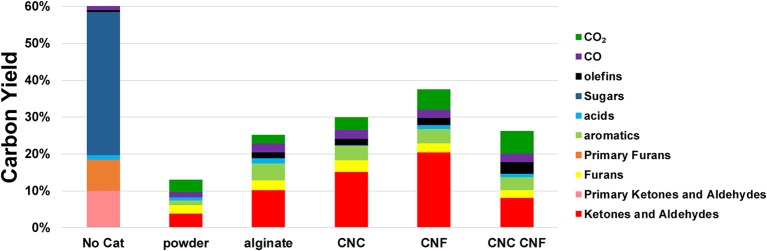
Carbon yields product classes produced by catalytic upgrading of cellulose pyrolysis vapors. Molecules classified as furans, ketones, and aldehydes observed in primary pyrolysis vapors are denoted as Primary Furans and Primary Ketones and Aldehydes and are composed of largely different species than those observed in the same classes after catalytic upgrading.

Quantification of the py-GC-MS/FID experiments allows for direct comparison of yields of various products from the templated catalyst and non-templated control. These results are presented in Figure 8 which also includes the yields from cellulose pyrolysis in the absence of a catayst for comparison. In these control experiments the carbon loss to char is ~40% due to the poor heating rates of the micropyrolyzer which is well documented in previous reports (e.g., Proano-Aviles et al., 2017). The difference between the char carbon yield and measured yield is due to catalyst coking and nonvolatile products which are not measured by GCMS. The total carbon yields of all of the catalytic pyrolysls experiments are lower than that of cellulose pyrolysis performed in the absence of any catalyst, which is expected due to coke formation and condendsation non-volatile products on the catlyst (Mukarakate et al., 2014). Each catalyst variant resulted in nearly complete conversion of the primary cellulose pyrolysis products. None of the of pyrolytic sugars (composed of mostly levoglucosan) present in the primary pyrolysis vapors were detected in the CFP experiments. Molecules classified as primary furans, ketones, and aldehydes were largely different species than those observed in the same classes after catalytic upgrading. Prior to catalytic upgrading the primary furanics observed were furan, 5-hydroxymethylfurfural, furfural, and furfuryl alcohol. After upgrading, partially deoxygenated and alkylated furanics were observed including furan (also observed in the primary pyrolysis vapors), 2-methyl-furan, 2,5-dimethyl-furan, 2-ethyl-furan. Molecules classified as ketones and aldehydes present in the primary pyrolysis vapors consisted mainly of formaldehyde, acetaldehyde, methyl glyoxal, 1-hydroxy-2-propanone, cyclopentanone, and 2-methyl-cyclopentanone. Following catalytic upgrading, the dominant ketone and aldehyde products observed were propenal, butanal, butanone, pentanone, cyclopentanone (which was also observed in the primary pyrolysis products), cyclopentenone, hexanone, cyclohexanone, and methylated variants of these molecules. The templated catalyst variants displayed some differences in yeild ehancments; however, all the templated catalysts produced appreciablely higher yeilds then the non-templated control for each class of products. Increases of ~100% were observed for ketone and aldeyde products for each of the templated catalysts. Yields of furanic compounds from the templated catalyts were similar on average to that of the non-templated control. Slight increases in furans were observed by the materials templated with alinate alone and CNC/CNF, while the other templated materials showed a reduction in furan production. Moderate improvements in aromatic yields were observed for all of the templated catalysts, ranging 10–50%. The increase in olefin production was the highest of any of the product classes analyzed at 100 to nearly 400% with respect to the non-templated control. Production of CO was also higher from all of the templated catalysts. Differences in CO_2_ production from the templated catalysts with respect to the control varied substantially, with CNF and CNF/CNC producing more the control, the alginate templated material producing less than the control, and the CNC templated material performing similar to the control.

### Extension of the Templating Method to Other Materials

**Figure 9:**
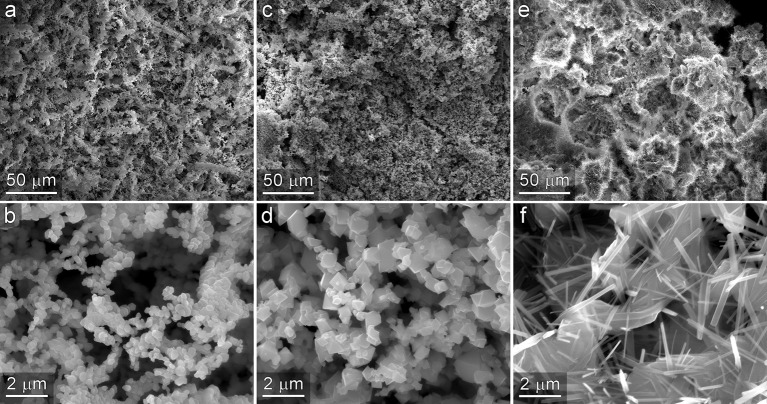
Templated materials prepared from NiCl_2_ **(a,b)**, FeCl_2_ **(c,d)**, and MgCl_2_ **(e,f)**. In each case the resultant material exhibits macroporosity observed in the low magnification images shown in the top row, and a 3-dimensional network of interconnected micro and nanoscale structures shown in the bottom row.

To investigate the generality of the NC-based templating method to other oxide materials, we performed the templating process using a mixture of alginate, CNF, and CNC while substituting several other metal chlorides, consisting of NiCl_2_, FeCl_2_, and MgCl_2_, for CeCl_3_. SEM micrographs of the resultant materials are presented in Figure 9. In each case, hiearhically structured materials were observed. Low magnification images shown in the top row of Figure 9 reveal high-surface area materials that contain macro porosity. Higher magnification images shown in the bottom panel of Figure 9 reveal that nanostructure of material consists of 3-dimensional interconnected system of micro and nanostructures. The nanoscale characteristics of each material are notably different from each other and reflective of the crystal structure formed by the oxide produced during the high temperature oxidation and template removal step. While comprehensive characterization and evaluation of catalytic activity of these new materials are beyond the scope of the current manuscript, these observations do provide evidence of the extensibility of the templating approach to other materials, and additional support for the assertion that the resultant structures consistently contain 3D interconnected networks of micro- and nanoscale dimensions.

## Discussion

Many templating strategies require the use of relatively expensive reagents or nanomaterials that make scaling to industrial demands prohibitive on the basis on cost and/or materials availability. In contrast, the low cost and vast production capacity of NC make it an ideal precursor for many industrial applications. While commercialization of NC is still in its infancy, the development of large volume industrial applications will provide the market pull to incentivize establishment of larger-scale production facilities to meet demands and further reduce the cost of NC.

In the context of CFP, the use of catalysts to increase the yield of desirable products from renewable feedstocks is critical. Some degree of deoxygenation of the primary pyrolysis vapors is necessary to achieve compatibility for blending and co-processing in existing petroleum refineries (Nolte and Shanks, 2017). However, recent studies have suggested that the additional reactivity of oxygenated biofuels may provide performance advantages over conventional hydrocarbon fuels in advanced engine formats, such as homogeneous compression ignition (HCCI) engines (Komninos and Rakopoulos, 2012). Additionally, the oxygenates produced by biomass deconstruction provide rich functionalities that can enable production of commodity chemical co-products. In many cases, biomass-based pathways may be a direct route to produce these oxygenates, while petroleum-based routes often require energy intensive and environmentally damaging oxidation of hydrocarbon precursors (Fitzgerald, 2017). These factors suggest that investigation of reducible metal oxide catalysts that promote formation of useful molecular functionalities from biomass deconstruction products, such as the ketones, aldehydes, and olefins produced by the CeO_2_ catalysts produced in the present study. The products obtained from both commercially available and templated CeO_2_ material in our experiments are largely expected from amphoteric metal oxides based on previous studies. Ketonization to form linear ketones has previously been reported (Mante et al., 2015) and can result from reactions of acids on the surface (Pham et al., 2013) or aldehydes (Gangadharan et al., 2010) with oxides on the surface to form carboxylate. Aldehydes and acids are commonly found in cellulose pyrolysis vapors and linear ketones, such as 2-butanone, 2-pentanone, and 3-pentanone were observed in the product streams from all of the ceria samples tested. Cyclopentenones are also observed from all of the ceria experiments, and these could result from ketonization reactions of diols or from ring opening of furfural (Omotoso et al., 2019). Extensive alkylation of furans, aromatics and cyclopentenones is also observed and likely results from the acid sites (Vivier and Duprez, 2010) on CeO_2_. These bimolecular reactions are strongly dependent on catalyst surface area, which explains the higher yields observed for the hierarchical ceria catalysts.

Among the templated catalysts, the total carbon yield was observed to be highest for the material templated by CNF and CNC, which were the two materials that contained the most mesoporosity. These results suggest that the mesoporosity may faciliate improved access to active sites throughout the catalyst as well as more rapid escape of products from the particle interior thereby reducing coke formation. CNF tend to form relatively large, high aspect ratio bundles in solution. An example of one such bundle observed via TEM is included in Figure S5. These structures likely give rise to macroporous channels throughout the material, which enables the higher overall carbon yield from the CNF-templated material by enabling escape of products prior to coke formation. Interestignly, the combination of CNC and CNF in the templating solution did not result in improved carbon yields with respect to the material templated by CNC alone. The origins of this result are not fully understand, however we suspect it may be attributed to the disruption of macro channel formation by CNCs. In addition to the increase in availability of active sites, the smaller average crystallite size present in the templated catalysts likely plays a substantial role in their improved activity. The work of Natile et al. (2005) demonstrated the improved reactivity of nanostructured CeO_2_. A recent perspective by Trovarelli and Llorca (2017) highlighted the importance of size, shape, and surface atomic arrangements of nanostructured CeO_2_ in determining its catalytic performance. The fingerlike nanoscale features exhibited by the templated catalysts synthesized in this study arise from steric hindrance by the carbohydrate templating materials. These semi-continuous nanostructures will likely result in the exposure of additional high-energy surfaces of CeO_2_ which will increase and modify its reactivity with respect to the bulk powder. The overall increase in volatile products from the templated catalyst observed in the py-GCMS experiments could results from the generally more active surface which accelerates reactions of polar pyrolytic sugars (i.e., levoglucosan) and subsequent release of products from the surface. Subtle modifications in the fractions of exposed crystal faces imparted by variations in the templating method may be responsible for the changes in product distribution exhibited by the various catalysts. The changes in total carbon yield and product distribution observed here result from a combination of reaction mechanisms, adsorption/desorption kinetics, and multiscale diffusion and possibly advection through the macropores. The lack of a detailed understanding of these phenomena is a limitation of the present study but should be the topic of future investigations in order to elucidate structure/function relationships and identify optimal synthesis targets for this templating approach.

While successful at introducing meso- and macroporosity to inorganic materials, this templating method does not produce specific surface areas as high as achieved in some previous reports of templating methods that result in mesoporous materials (Laha and Ryoo, 2003; Valentin et al., 2005; Shopsowitz et al., 2010). A potential route to improve the surface-area enhancement of the method described in the present work could be to combine the surfactants used in the aforementioned studies with the nanocellulose components employed here, thereby increasing the surface area by the introduction of more ordered mesoporosity while still maintaining the macroporous channels imparted by the nanocellulose. Another potential means to improve the surface area could be optimization of the drying and template removal process, which has been shown previsouly to have a large impact of the resultant structure of carbhydrate-based structures (Valentin et al., 2005).

## Conclusions

This simple, inexpensive, scalable, synthesis procedure allows for templating metal oxide structures using renewable, bio-derived materials to yield tunable porosity and morphology. In comparison to CeO_2_ powder, catalysts synthesized with alginate and nanocellulose templates showed a nearly 10-fold enhancement in surface area and increased meso- and macroporosity. When used for catalytic upgrading of cellulose fast pyrolysis vapors, CeO_2_ material produced from carbohydrate templates exhibited substantially higher catalytic activity than an equivalent mass of non-templated CeO_2_ powder. Several variations of alginate, CNC, and CNF templates were produced and evaluated. Each templated variant substantially outperformed the non-templated control in CFP experiments, and it was observed that the product distribution was impacted by variations in the catalyst architecture resulting from the different compositions of the carbohydrate templating suspensions. Finally, we demonstrated that this templating methods could be extended materials synthesized from other metal chloride precursors to achieve 3-dimensional networks of hierarchical porosity. Cost-effective routes to increasing the functionality of metal oxide catalysts as demonstrated here can potentially improve the economic feasibility of biomass conversion strategies. Overall, these results demonstrate that nanocellulose can serve as an effective templating agent by introducing controlled porosity and morphology to enhance surface area and introduce higher order architecture within catalyst particles.

## Author Contributions

Study was conceived by PC, KM, and MN. Catalyst synthesis was performed by KM and PC. Catalyst characterization was performed by KM, DC, DV, and PC. CFP experiments were performed by CM. Manuscript text and figures were prepared by KM and PC with assistance from all authors.

### Conflict of Interest

The authors declare that the research was conducted in the absence of any commercial or financial relationships that could be construed as a potential conflict of interest.
