# Supplementary material for: Hierarchically Structured CeO2 Catalyst Particles From Nanocellulose/Alginate Templates for Upgrading of Fast Pyrolysis Vapors
Source: Front Chem. 2019 Oct 30;7:730. doi: 10.3389/fchem.2019.00730 (PMC6831546; doi:10.3389/fchem.2019.00730)
Supplement: Supplementary file 1 [file Data_Sheet_1.PDF]

Supplemental Materials for:

Hierarchically structured CeO_2 catalyst particles from nanocellulose/alginate templates for upgrading of fast pyrolysis vapors

Kathleen Moyer,[1] Davis Conklin,[2] Calvin Mukarakate,[2] Derek Vardon,[2] Mark Nimlos,[2] and Peter N. Ciesielski[3,*]

[1]Interdisciplinary Materials Science Program, Vanderbilt University, Nashville, TN 37235

[2]National Bioenergy Center, National Renewable Energy Laboratory, Golden CO 80401

[3]Biosciences Center, National Renewable Energy Laboratory, Golden CO 80401

*Correspondence to: peter.ciesielski@nrel.gov

S1. Thermal gravimetric analysis of template removal

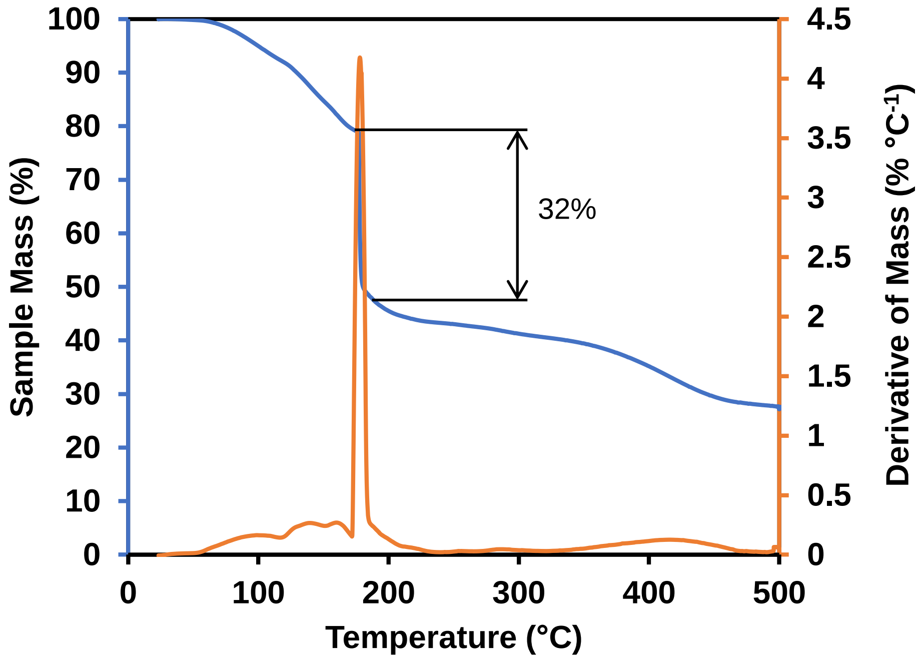

Figure S1. Thermal gravimetric analysis of template removal during the high temperature oxidation process.

To confirm complete removal of the carbohydrate template, TGA was performed on the CNC-CNF templated sample in a static air environment to emulate the calcination step of catalyst synthesis. The major 32% mass loss around 170-190°C is attributed to removal of the bulk of the templating material. An additional isothermal hold for 1 hour at 500°C was appended to the TGA method, during which the mass changed by less than 2%.

S2. Variations in CeO2 structure from different preparation methods

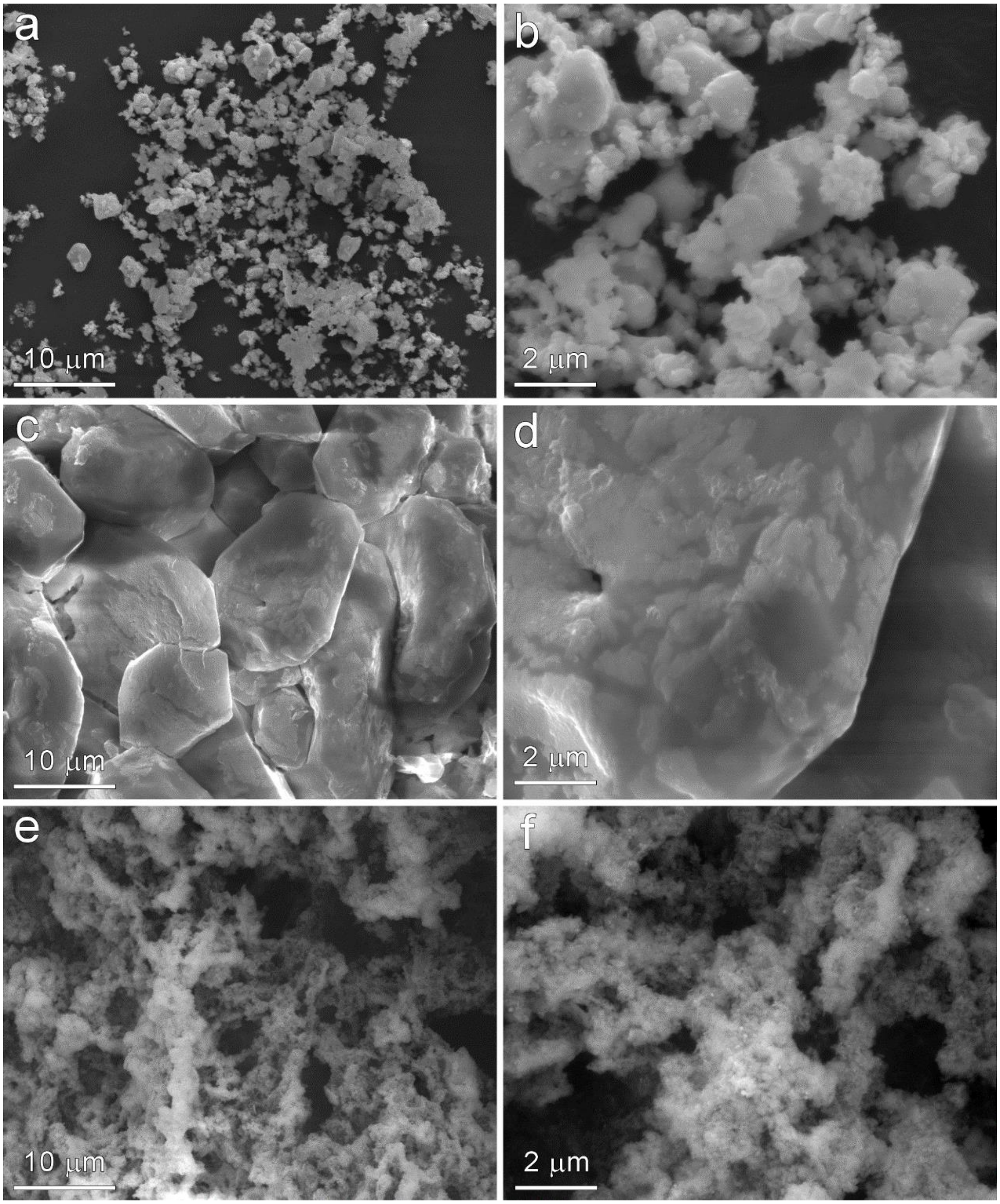

Figure S2. SEM micrographs of commercially available CeO_2 powder (a, b), CeO_2 obtained by freeze drying in the absence of a template followed by high temperature oxidation (c, d), and CeO_2 obtained by the templating process described in section 2.1 of the main text.

In order to further investigate the impacts of the templating procedure, a CeO_2 control sample was prepared by freeze-drying a solution containing $CeCl_3$ and subjecting the resultant material to the same high-temperature oxidation process that was used to remove the carbohydrate from the templated materials. Nitrogen physisorption and BET analyses were performed to estimate the specific surface are and mesopore volume of the freeze-dried material to be 7 m^2/g and 0.0259 cm^3/g, respectively. Figure S2 presents SEM micrographs comparing the commercially available CeO_2 powder (Figure S2a and b), the CeO_2 control prepared without a template (Figure S2c and d), and CeO2 prepared with the alginate/CNF/CNC template (Figure S2e and f). The non-templated material exhibits agglomerates of crystallites with sizes on the order of several to tens of microns which appear larger than those present in the commercial CeO_2 powder. The templated material clearly exhibits larger channels and finer nanoscale features than both commercial CeO_2 powder and the material produced without a template. These observations support the assertion that the increase in surface area in the templated materials indeed originates from the carbohydrate template rather than the freeze-drying process.

S3. t-plot analysis

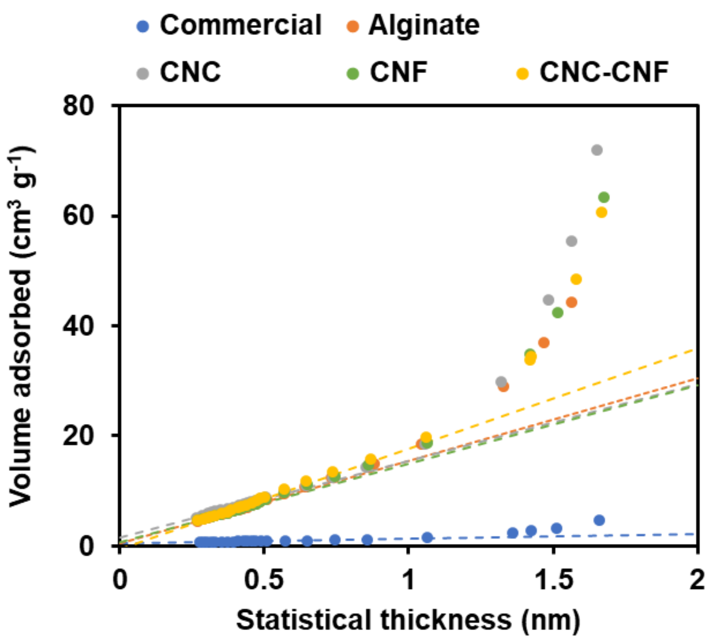

Figure S3. t-plot analysis of the N2 physisorption results verify that both the commercial and templated catalysts have negligible micropore volume.

S4. EDS analysis of the templated CeO_2 material

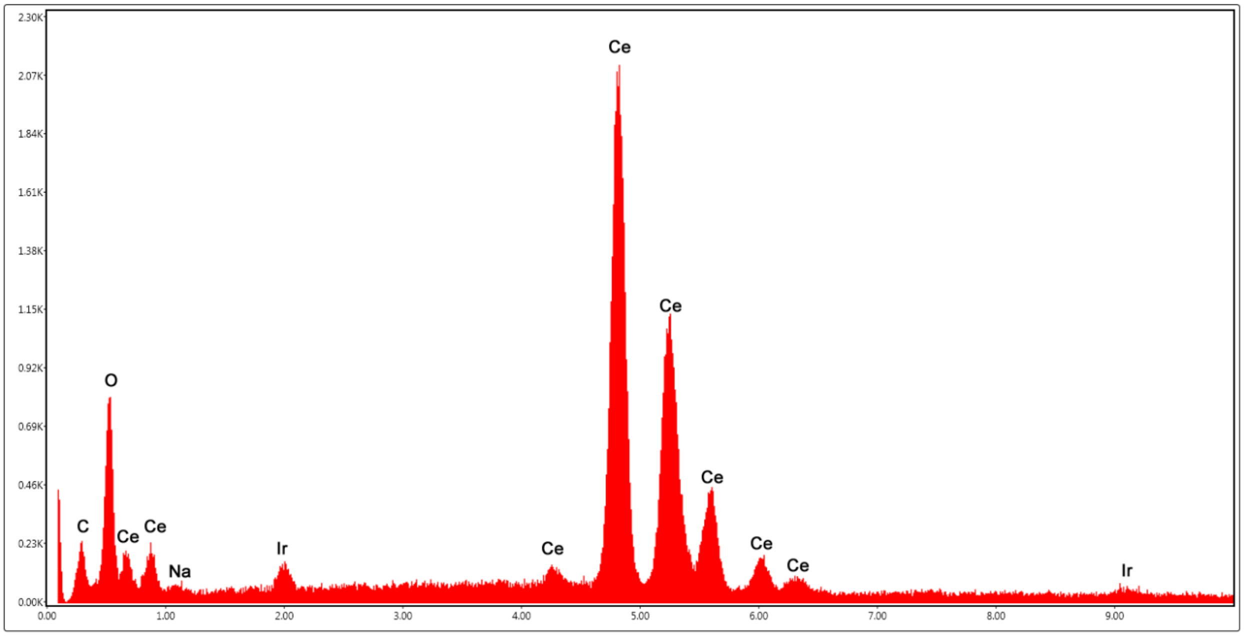

Figure S4. EDS spectrum of a templated catalyst particle.

Table S1. Elemental composition of a templated catalyst particle measured by EDS.

Element	Weight %	Atomic %
C	6.7	24.61
O	18.56	51.14
Na	0.62	1.18
Ce	71.11	22.38
Ir	3.01	0.69

EDS was performed to assess the elemental composition of the templated catalysts. A representative EDS spectrum obtained from a catalyst templated by a mixture of alginate, CNF, and CNC is presented in Figure S1. The elemental composition obtained by quantitative analysis of the data is shown in Table S1. The highest intensity peaks in the spectrum correspond to Ce and O as expected. The sample was sputter coated with Ir in preparation for SEM imaging prior to EDS analysis which accounts for the presence of this element in the spectrum. The carbon signal

is primarily attributed to the carbon tape which was used to mount the sample on the SEM stub. A very small amount of Na was observed in this sample. This element served as the original counter ion for the negatively charged alginate polymer present in the carbohydrate template. It's presence within the templated material indicates that it was not completely displaced upon addition to the $CeCl_3$ solution. Quantifying the impact, if any, of the presence of this contaminant upon catalyst performance is beyond the scope of the present work but should be the topic of future investigations.

S5. TEM images of CNF bundles

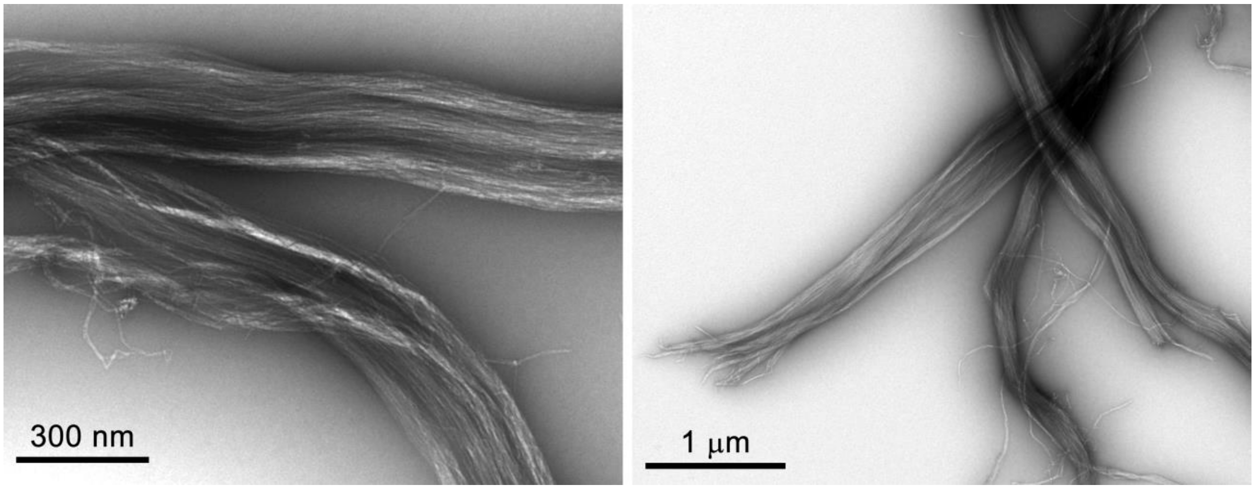

Figure S5. Additional TEM images of large CNF bundles.

The CNF used in this study tend to form some population of large bundles with diameters on the order of hundreds of nanometers or larger. These structures likely give rise macroporous channels throughout the materials obtained by templates including CNF.
